# A Novel Candidate for Guided Tissue Regeneration: Chitosan and Eggshell Membrane

**DOI:** 10.7759/cureus.48930

**Published:** 2023-11-16

**Authors:** Shubhangini Chatterjee, Kaarthikeyan G

**Affiliations:** 1 Periodontics, Saveetha Dental College and Hospitals, Saveetha Institute of Medical and Technical Sciences (Deemed to be University), Chennai, IND

**Keywords:** guided tissue regeneration, membrane, characterization, chitosan, eggshell

## Abstract

Background

The primary cause of adult tooth loss is commonly attributed to periodontal disease, a condition that weakens the supportive structures around the teeth. In addressing periodontal diseases, surgeons often employ the guided tissue regeneration (GTR) technique, which involves the use of a barrier membrane.

Aim

The aim of the present study is to assess the composition and mechanical strength of chitosan and eggshell membrane. This research was conducted to provide insights into their potential application in facilitating tissue regeneration.

Materials and procedures

Chitosan and eggshell membrane were combined to create the membrane. Scanning electron microscopy (SEM) using FEI Quanta FEG 650 SEM (JSM IT-800, JEOL Ltd., Akishima, Tokyo, Japan) was carried out, and mechanical properties were used to measure the parameters of membrane characterization.

Results

In the dry condition, the membrane's tensile strength was 0.30 MPa and its elongation at break was 8.2%. In the wet condition, the membrane's tensile strength was 0.13 MPa and its elongation at break was 22.6%. The SEM results depicted membrane surface with pore sizes ranging from 16 to 100 meters, and the result obtained from membrane porosity test was 31.2%.

Conclusion

The chitosan-eggshell membrane exhibited a fibrous surface with a desirable pore size for use as a GTR membrane, but it has low mechanical strength.

## Introduction

Globally, a significant portion, ranging from 20% to 50%, of the population grapples with the prevalence of periodontal disease, a consequential challenge that adversely impacts oral health. This condition stands as the predominant factor contributing to the loss of adult teeth. However, a substantial challenge persists in the realm of rectifying the damage inflicted upon periodontal tissue and the accompanying bone support. In contemporary dental medicine, the concept of "restitutio ad integrum", a Latin term meaning restoration to original condition, takes the forefront, representing a modern approach, particularly within the sphere of dental implantology. In the current landscape, the emphasis has shifted toward prioritizing prevention over treatment. This shift underscores a significant change in perception and approach toward medical practices [1]. An increasing number of patients are seeking comprehensive dental rehabilitation that encompasses fixed solutions, eliminating the need for pink veneering porcelain or other synthetic prosthetics. The presence of substantial alveolar bone resorption can pose a barrier to the successful placement of dental implants, thereby complicating the recuperation process considerably. Consequently, the necessity for alveolar bone augmentation procedures arises frequently, albeit these interventions carry inherent risks and the potential for adverse effects.

The field of biomaterials is undergoing rapid expansion in relation to regeneration, with a heightened focus on research endeavors aimed at pinpointing the optimal process for regeneration [2]. An ideal material must satisfy specific prerequisites such as biocompatibility and user-friendliness as well as possess bone-inductive and bone-conductive properties [3]. In addition to the aforementioned criteria, antibacterial attributes, affordability, and the presence of viable cells capable of bone regeneration may also be essential attributes for the biomaterial [4].

The guided tissue regeneration (GTR) technique employs a space-preserving barrier membrane to facilitate the adequate migration, proliferation, and maturation of periodontal ligament and bone cells. This approach effectively hinders the swift migration of connective tissues and gingival epithelial cells, known for their rapid growth, into the affected region, enabling the intended regenerative process [5]. Over the last few decades, a diverse array of GTR membranes have been developed, encompassing both non-resorbable and bioresorbable options. Non-resorbable membranes, like expanded polytetrafluoroethylene (e-PTFE), exhibit superior mechanical strength and are capable of encompassing larger areas. However, acquiring these materials requires a distinct surgical procedure due to their non-degradable nature [6]. As a result, there has been a noticeable rise in the utilization of bioresorbable GTR membranes. These membranes are predominantly composed of synthetic polymers such as polylactic acid (PLA) and natural biopolymers like collagen. While collagen membranes are often subject to criticism due to their compromised mechanical attributes, unpredictable degradation rates, and relatively higher manufacturing costs and the intricate nature of their manufacturing processes, they excel in terms of superior biocompatibility and affinity for cells [7]. Two significant drawbacks associated with synthetic bioresorbable membranes are their limited biocompatibility and their potential to induce inflammation [8]. Considering the existing challenges posed by the membranes available in the market, there remains a crucial need to develop an ideal barrier membrane that effectively addresses the requirements for biocompatibility and the ability to create space. This development is essential to ensure the consistent and predictable regeneration of periodontal tissues in clinical settings.

The natural eggshell membrane (ESM) constitutes a dual-layered, non-calcifying structure situated between the egg albumin and its inner shell surface. Comprising interwoven protein fibers that resist water solubility, ESM exhibits an intricate network, predominantly bound together by numerous disulfide bridges [9,10]. It also serves a pivotal function in the subsequent process of eggshell biomineralization [11,12], and the complete process of eggshell biomineralization transpires in less than 24 hours.

Chitosan (poly-1,4-D-glucosamine) is a common alkaline polysaccharide distinguished by its positive charge. Derived from deacetylated chitin, it is notable for its remarkable biodegradability, biocompatibility, and minimal toxicity [13]. Furthermore, chitosan has received considerable recognition in recent years due to its ease of use in creating nanofibers, gels, scaffolds, membranes, and nanoparticles [14].

The aim of this study is to create a GTR membrane utilizing chitosan and ESM and subsequently assess the membrane's characteristics, including its elongation at break, morphology, and tensile strength. 

## Materials and methods

Materials and tools used for the fabrication of membrane were as follows: ground eggshells, chitosan, distilled water, 1% acetic acid, a cross-linker, tripolyphosphate (TPP) solution, sodium hydroxide, Petri dishes, a universal testing machine, and a scanning electron microscope.

Eggshell and chitosan membrane fabrication

In the experimental process, 0.2 g of chitosan was dissolved in 20 ml of distilled water. To this solution, 400 μL of 1% acetic acid was added, and the mixture was stirred for a period of 30 minutes. Subsequently, a cross-linker called N-(3-dimethylaminopropyl)-N-ethylcarbodiimide hydrochloride was introduced. Following this, 1 g of finely ground eggshell powder was incorporated into the solution at two distinct concentrations: 5% and 10%. The resulting chitosan-eggshell solution was subjected to vigorous stirring, while a 2% (w/v) aqueous TPP solution was gradually added drop by drop. Following 10 minutes of stirring, the resulting mixture was subjected to centrifugation at 1,500 revolutions per minute (rpm) for 30 minutes. Post centrifugation, the mixture was poured into Petri dishes, and the resultant material was thoroughly rinsed with distilled water multiple times and then subjected to the process of freeze-drying. The chitosan-ESM obtained was then immersed in a solution of sodium hydroxide (NaOH), followed by multiple distilled water washes until the pH of the membrane reached neutrality. The mixture was then lyophilized and frozen to yield the chitosan-ESM. In parallel, the same methodology was applied to produce pure eggshell and chitosan membranes. The morphology of these membranes was examined using scanning electron microscopy (SEM). This assessment encompassed observing the cross-section for both the 5% and 10% concentration variations.

Eggshell and Chitosan Membrane Characterization

SEM was employed for analysis. The samples were placed and assessed using the FEI Quanta FEG 650 SEM (JSM IT-800, JEOL Ltd., Akishima, Tokyo, Japan), operating at an accelerating voltage of 2,000 kV. Photographs were captured of both the membrane's surface and cross-sections, at magnifications of 100× and 500×. It can be employed for imaging samples with a resolution of up to 0.5 nm. Additionally, it comes equipped with an assortment of detectors suitable for tasks such as elemental mapping, crystallographic analysis, and more.

Tensile strength measurement: In order to assess the mechanical properties, the membrane was cut into small pieces, and then the cut ends were affixed to the testing apparatus. The tensile load was expressed in newtons (N). Subsequently, the chitosan-ESM was subjected to gradual tension at a specified rate until it fractured. The recorded data consisted of the tensile load at the moment of fracture. This testing procedure was carried out using a universal testing machine.

## Results

Characterization of chitosan-ESM

Chitosan was combined with eggshell to fabricate a membrane; the fabrication was performed at the White Lab, Saveetha Dental College and Hospitals, Chennai. The produced membranes were sliced into pieces measuring 1 × 1 cm and underwent sterilization. Subsequently, various assessments, including elongation at break, membrane porosity, tensile strength, and SEM analysis, were carried out.

Tensile strength is the maximum stress a material can endure when stretching or pulling forces are applied before it fractures. Assessment of the elongation at break and tensile strength of the membrane was done. In the dry state, the chitosan-ESM exhibited a tensile strength of 0.30 MPa, and the elongation at break was recorded at 8.2%, whereas in the wet state, the tensile strength measured 0.13 MPa, and the elongation at break was recorded at 22.6% (as depicted in Table 1).

**Table 1:** Tensile strength and elongation at break The tensile strength and elongation at break of the prepared chitosan-eggshell membrane

Membrane condition	Tensile strength (MPa)	Elongation at break (%)
Dry	0.30	8.2
Wet	0.13	22.6

Porosity testing quantifies the spaces within a material, which is a fraction of the volume of voids to the total volume, and range is 0-100%. In this study, the chitosan-ESM had a porosity result of 31.2%, indicating that approximately 31.2% of the membrane's total volume consisted of voids or spaces.

**Figure 1:**
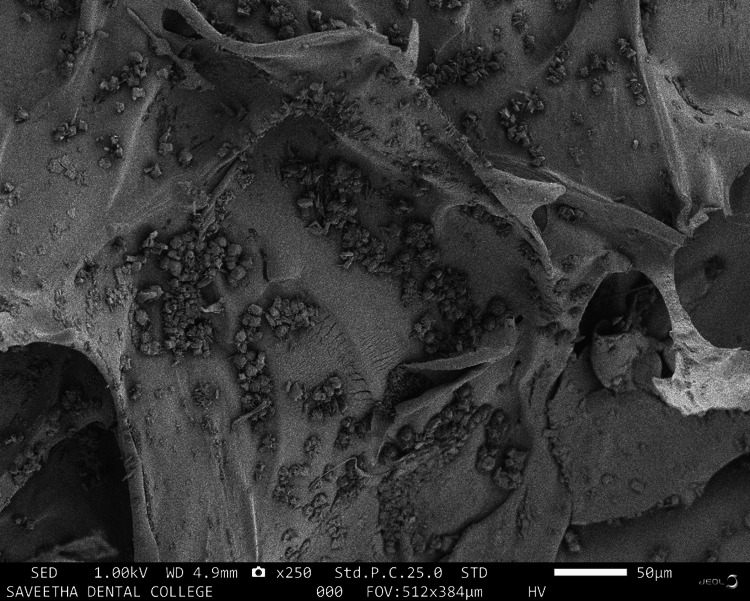
SEM analysis of the chitosan-eggshell membrane The SEM analysis of the chitosan-eggshell membrane revealed distinct characteristics at different magnifications. At 250× magnification, the membrane exhibited a porous surface. SEM: scanning electron microscopy

**Figure 2:**
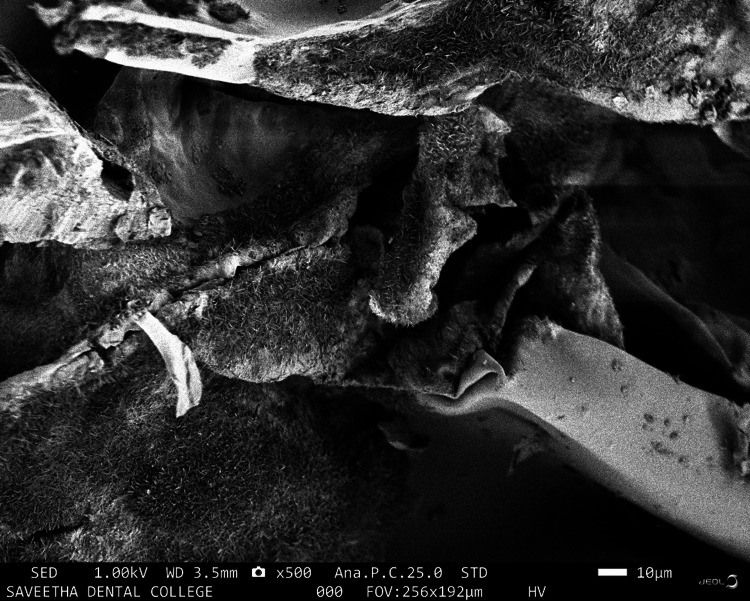
SEM analysis of the chitosan-eggshell membrane When observed at 500× magnification in a cross-sectional cut, the fibrous and interconnected porous structure was clearly discernible. SEM: scanning electron microscopy

SEM is designed for direct surface observation of solid objects. The SEM analysis of the chitosan-ESM revealed that eggshell elements effectively integrated with chitosan, resulting in a homogeneous mixture. Additionally, the membrane's surface displayed pores of varying sizes, ranging from approximately 16 μm to 100 μm (as depicted in Figure 1 and Figure 2).

## Discussion

Based on the results obtained in this study, it can be concluded that the chitosan-ESM displays a surface distinguished by its fibrous texture. The membrane also demonstrates interconnected porosity, coupled with an optimal pore size suitable for its intended purpose as a GTR membrane. However, it's worth noting that the mechanical strength of the membrane is comparatively lower. 

Chitosan is recognized as one of the most extensively studied natural biomaterials and has garnered significant interest for its potential as a material for GTR membranes. Nevertheless, certain reports have raised apprehensions regarding the mechanical and biological attributes of chitosan. These concerns include its limited bioactivity and insufficient rigidity, especially when it comes to its resorbable properties, particularly in wet conditions. Numerous studies have been conducted with the objective of enhancing chitosan's characteristics by blending it with other organic or inorganic materials to create composite or hybrid materials.

The effectiveness of combining two polymer components depends on the interactions taking place at the molecular level. These interactions culminate in the enhancement of mechanical properties within the alloy. Parameters such as elongation at break and tensile strength measure the strength of the membrane and its flexibility. These physical attributes are of paramount significance, exerting a pivotal role in validating the membrane's viability for clinical applications. On the basis of our findings depicted in Table 1, it was revealed that the fabricated membrane exhibited a tensile strength of 0.30 MPa in dry conditions, whereas it was 0.13 MPa in wet conditions. The relatively low tensile strength observed can be attributed to the fact that the primary composition of this membrane consists solely of natural materials, without the incorporation of synthetic chemicals aimed at augmenting its mechanical attributes. Compared to certain membranes on the market, our membrane exhibits a lower tensile strength. For instance, the Bio-Gide membrane (Geistlich Pharma AG, Wolhusen, Central Switzerland) boasts a maximum tensile stress of 4.8 MPa and a maximum tensile strain of 46.8%. Likewise, the collprotect membrane (botiss biomaterials GmbH, Zossen, Brandenburg, Germany) demonstrates a maximum tensile stress of 13.1 MPa and a maximum tensile strain of 13.1% [15].

Studies using animal models showed that bioceramic scaffolds are useful in bone regeneration procedures. The material's architecture holds the key to ensuring proper bone healing. It should have a networked, porous structure resembling natural cancellous bone to promote cell ingrowth, proliferation, and differentiation. In functionally loaded regions like the alveolar bone, the biomaterial must possess the requisite mechanical properties [16]. The biomaterial's rate of resorption needs to match the rate of osteogenesis developing in the new bone [17]. Utilizing composite materials in conjunction with biodegradable polymers is one method of altering these rates [18].

In a study, it was indicated that bone marrow stromal cells (BMSCs) demonstrated robust growth and proliferation when cultured on natural ESM [19]. As a result, it was inferred that ESM could serve as a viable scaffold material for bone tissue engineering [19]. These studies found that ESM has good biocompatibility, a fibrous topology, and the ability to improve tissue healing, implying that it could potentially be a good candidate for GTR membrane. ESM is also widely available as a byproduct of the food processing industry. In 2007, global egg production was 62.5 million tonnes, and egg consumption in the United States was 6.53 billion pieces [20]. The reuse of these discarded eggshells would obviously be both environmentally and economically beneficial. Some researchers have attempted to discover this potential in animal studies by using natural ESM as a GTR membrane [21,22]. However, the results were not satisfactory due to the poor space-maintaining ability and early collapse of the membranes, suggesting that further studies were essential to improving these functions.

The physical attributes of a scaffold hold paramount importance in the realm of tissue engineering. It's imperative for this scaffold structure to exhibit elevated porosity and interconnected porous configurations. These features are integral in facilitating essential cellular functions such as attachment, proliferation, and differentiation [23]. In previous researches, non-chitosan-coated membrane exhibited a larger porous size spanning 30-100 µm, while the chitosan-coated membrane demonstrated relatively smaller pores spanning 30-70 µm. This variation in porous size and distribution underscores the impact of chitosan coating on the resulting membrane structure. Both collagen- and chitosan-based membrane or scaffold possessed interconnected porous structures, exhibiting an average diameter spanning 75-150 µm [24]. Notably, a minimum pore size of 100 µm has been cited as the recommended threshold for scaffold structures to achieve sufficient vascularization for tissue or organ repair and regeneration, as previously reported [25].

In line with this, the SEM analysis depicted in Figure 1 and Figure 2 reveals a homogeneous blending of chitosan and ESM elements. Furthermore, it's worth noting that the membrane's surface porosity exhibits a range of sizes, typically spanning from around 16 µm to 100 µm. This configuration aligns with the prerequisites for optimal cell interactions and tissue regeneration. The creation of a porous scaffold structure is intricately influenced by the freeze-drying technique employed. In the course of freezing, ice grains come into being. The membrane, featuring an interconnected porous surface, serves to enhance crucial cellular processes such as attachment, proliferation, and differentiation, which are pivotal for both wound healing and tissue regeneration. Due to their varied roles in cell attachment, growth, and tissue regeneration, chitosan is gaining prominence in clinical applications. 

Periodontal treatment is aimed at effectively resolving inflammation and achieving the regeneration of tissues [26]. An effective barrier membrane must adhere to critical design criteria, with biocompatibility being a primary consideration. It is imperative that the membrane does not trigger an immune response or induce sensitization, which could be detrimental to wound healing. In order to prevent undesirable cells from moving toward the material, the membrane should function as a barrier for these cells. Nonetheless, it should be able to allow the nutrients and gases to pass through in order to support the regenerative process. Tissue integration is another pivotal characteristic of a barrier material. The characteristic of being able to establish a space adjacent to the root surface is important and enables cells from the periodontal ligament to occupy this space, thus promoting the regrowth of tissues. Furthermore, the membrane's design should facilitate easy trimming and adjustment at the intended site, ensuring practicality and precision during application.

Low mechanical strength in a GTR membrane can compromise its effectiveness in several ways. Handling and placement difficulties during surgery, inadequate conformability to tissue contours, premature degradation, compromised barrier function, and instability at the implantation site are key concerns. A weak membrane may fail to protect regenerating tissue from unwanted cell ingrowth, impacting its ability to guide proper tissue regeneration. Additionally, low mechanical strength can hinder cell adhesion and migration, essential processes for successful tissue healing. Ultimately, addressing these issues through material optimization and design considerations is crucial for ensuring the membrane's structural integrity and overall effectiveness in facilitating GTR.

To enhance the mechanical strength of the novel chitosan-ESM for GTR, employing strategies such as cross-linking with agents like glutaraldehyde, blending with polymers like polyvinyl alcohol, incorporating reinforcing materials such as nanoparticles or natural fibers, utilizing electrospinning for nanofibrous structures, optimizing processing parameters, and applying heat treatment cautiously can be of advantage. Additionally, layering or coating the membrane and experimenting with different chitosan characteristics further refine mechanical performance. Comprehensive biocompatibility testing is crucial to ensure these modifications do not compromise the safety and effectiveness of the membrane in GTR applications. These methods aim to improve the overall structural integrity and performance of the membrane. However, it's also crucial to validate the biocompatibility of the modified membrane and collaborate with experts in biomaterials for further insights and guidance.

## Conclusions

Taking into account the constraints of this study, the findings lead to the conclusion that chitosan-ESM exhibit a fibrous surface characteristic and possess the recommended pore size suitable for GTR membrane applications. Despite its observed lower mechanical strength, this novel membrane stands out as a promising and environmentally conscious option, and it holds promise as a potential alternative for GTR barrier membranes. It is clear that future studies are necessary to improve the strength of chitosan-ESM, thus facilitating their ongoing development and utilization.
